# Family cluster of three cases of monkeypox imported from Nigeria to the United Kingdom, May 2021

**DOI:** 10.2807/1560-7917.ES.2021.26.32.2100745

**Published:** 2021-08-12

**Authors:** Gemma Hobson, James Adamson, Hugh Adler, Richard Firth, Susan Gould, Catherine Houlihan, Christopher Johnson, David Porter, Tommy Rampling, Libuse Ratcliffe, Katherine Russell, Ananda Giri Shankar, Tom Wingfield

**Affiliations:** 1 Health Protection, Public Health Wales, Cardiff, United Kingdom; 2 Tropical and Infectious Disease Unit, Liverpool University Hospitals NHS Foundation Trust, Liverpool, United Kingdom; 3 Departments of International Public Health and Clinical Sciences, Liverpool School of Tropical Medicine, Liverpool, United Kingdom; 4 Rare and Imported Pathogens Laboratory, Public Health England, Porton, Salisbury, United Kingdom; 5 Department of Virology, University College Hospital London, London, United Kingdom; 6 Department of Paediatric Infectious Diseases, Alder Hey Children's NHS Foundation Trust, Liverpool, United Kingdom; 7 Hospital for Tropical Diseases, University College London Hospitals NHS Foundation Trust, London, United Kingdom; 8 Emerging Infections and Zoonoses Section, National Infection Service, Public Health England, Colindale, London, United Kingdom; 9 World Health Organization Collaborating Centre on Tuberculosis and Social Medicine, Department of Global Public Health, Karolinska Institute, Stockholm, Sweden

**Keywords:** Monkeypox, health protection, Wales, England, Orthopox, Nigeria

## Abstract

Most reported cases of human monkeypox occur in Central and West Africa, where the causing virus is endemic. We describe the identification and public health response to an imported case of West African monkeypox from Nigeria to the United Kingdom (UK) in May 2021. Secondary transmission from the index case occurred within the family to another adult and a toddler. Concurrent COVID-19-related control measures upon arrival and at the hospital, facilitated detection and limited the number of potential contacts.

Monkeypox is a rare viral zoonotic disease. The causing virus belongs to the *Orthopoxvirus* genus that includes variola virus (the cause of smallpox), vaccinia virus (used in the smallpox vaccine), and cowpox virus. There are two distinct clades of monkeypox virus—Central African and West African. The first human cases were identified in the Democratic Republic of Congo in 1970 [1]. Outside of Africa, cases of human monkeypox infections have been documented in four countries: four cases the United Kingdom (UK) in 2018/2019, one case in Israel in 2018 and one case in Singapore in 2019 [2], 47 cases in the United States (US) in 2003 and one in 2021 [3]. We report on a family cluster of three recent cases of monkeypox in the UK associated with travel from Nigeria.

## Case description

In May 2021, the index case (Case 1) travelled with their family from Delta State, Nigeria to Manchester, UK via Istanbul, Turkey. They were asymptomatic while travelling. In line with the UK coronavirus disease (COVID-19) guidance, the entire family spent 10 days in isolation in a self-contained accommodation following their arrival to the UK [4].

Two days after arrival, Case 1 developed a vesicular lesion, with further vesicles developing over the next week while the entire family continued to self-isolate. Twelve days later, Case 1 presented at a local emergency department. They were reviewed in a hospital emergency department's COVID-19 zone (because of their recent travel history) by staff wearing personal protective equipment (PPE) for the care of COVID-19 patients (i.e. disposable gloves, apron, eye protection, fluid resistant surgical mask type IIR) [5], and discharged the same day. However, on the day after, following discussion between the emergency department and the UK Imported Fever Service, Case 1 was transferred to a high consequence infectious diseases (HCID) Unit in Liverpool, England. Based on urine samples and skin swabs collected on that day, Public Health England’s Rare and Imported Pathogens Laboratory confirmed 2 days later, by PCR and sequencing, the West African clade of monkeypox virus.

Concurrently, Case 1’s spouse and four young children were placed under active surveillance at their accommodation by Public Health Wales (PHW) supported by paediatric infectious diseases specialists. 19 days after Case 1 symptoms’ onset (ASO), the youngest child (18 months, Case 2) developed lesions compatible with early monkeypox; the decision was made to transfer the spouse and all four children to the same HCID Unit in Liverpool as Case 1 to enable clinical assessment of the family members. On day 21 ASO (day 2 after symptom onset in Case 2), PCR testing of the lesion swabs confirmed monkeypox virus infection and the toddler was managed with specialist paediatric support from the regional children’s hospital in Liverpool.

Meanwhile, by day 19 ASO, Case 1 was well, afebrile, and all skin lesions had crusted. They tested negative for monkeypox virus by PCR in urine, blood, swabs of lesions/lesion fluid and nose/throat swab samples on two occasions, 24 h apart. The three children with no features clinically compatible with monkeypox remained with Case 1 for monitoring and remained asymptomatic. On day 25 APO, Case 1 was discharged with the three children who did not develop monkeypox while under active surveillance within the 21-day follow-up period.

On day 33 ASO of Case 1, the other adult member of the family (Case 3), who cared for the Case 2 and resided in the same isolation room for the duration of their hospital admission, developed a vesicular rash. On day 3 after symptoms onset in Case 3, PCR of a lesion swab confirmed monkeypox virus. On days 24 and 10 of their respective symptom onset, Cases 2 and 3 clinically recovered and were discharged after confirmation of viral clearance. They tested negative for monkeypox virus by PCR in blood samples, urine samples and swabs of lesions/lesion fluid and nose/throat on two occasions, 24 h apart.

## Public health response

On notification of a probable and then confirmed case of monkeypox, the health authorities activated the incident management team and the HCID network. In accordance with the International Health Regulations (IHR) [6], information was reported to the World Health Organization (WHO) and the Nigerian national IHR focal point. Public Health Wales identified and contacts and stratified them by risk level. Contacts included household contacts, healthcare workers, hospital laundry workers, and members of the public who may have been exposed. Thirty contacts in Wales were identified for active surveillance and eight for passive surveillance [7]. Since Case 1 developed symptoms after arrival in the UK, aeroplane passengers on the incoming flight were discounted from surveillance.

Passive surveillance was used for individuals identified as having a low-risk exposure to Case 1, their body fluids, or potentially infectious materials. Low-risk exposure was considered to be physical contact with the confirmed case while wearing PPE (i.e. disposable gloves, gown and a filtering face piece FFP3 face mask). These individuals were provided with written information including phone numbers to contact PHW if they developed symptoms. Active surveillance was used for individuals identified as having an intermediate- or high-risk exposure to Case 1 (Cases 2 and 3 were already in isolation before becoming infectious), their body fluids, or potentially infectious materials. Direct exposure of broken skin or mucous membranes to a symptomatic monkeypox case, their body fluids or potentially infectious material (including on clothing or bedding) without wearing full PPE (including FFP3 or equivalent) was considered to constitute high-risk exposure. The PHW health protection team contacted these individuals daily throughout the 21-day follow-up period to check for potential prodromal monkeypox symptoms including fever, headache, myalgia, backache, lymphadenopathy, chills or exhaustion. No contacts travelled outside of the UK following exposure. All contacts identified for active surveillance completed the 21-day surveillance period from their last date of exposure. No transmission outside the index family was identified.

The local authority facilitated decontamination of the family’s residential accommodation by a UK government approved contractor. Post-exposure vaccination was not offered to contacts or the family members due to the level of exposure and late presentation of Case 1 at the hospital. Healthcare workers in the HCID Unit were offered vaccination with modified vaccinia Ankara (MVA-BN/Imvanex) vaccine.

## Discussion

Monkeypox was first discovered in 1958 [8]. It was not recognised as a human infection until 1970 during the smallpox eradication programme [9]. The largest outbreak reported outside of Africa was in the US where cases were found to be connected to imported pet rodents. More recent imported cases have all been associated with travel from Nigeria [2]. Since 2017, Nigeria has recorded its largest ongoing outbreak of monkeypox with a total of 446 cases and eight deaths [10].

The animal reservoir of monkeypox remains unknown, however, evidence suggests it may be rodents, including giant Gambian rats (*Cricetomys gambianus*) and squirrels [11]. Transmission through direct or indirect contact with live or dead animals, including through bushmeat consumption, hunting, or trade, is presumed to be the main factor for human monkeypox infections [12]. Human-to-human transmission is less common but possible through close contact with an infected person’s skin lesions, large respiratory droplets exhaled during extended face-to-face contact, or contaminated objects [2,8]. Genetic sequencing confirmed the cases described here as being West-African clade, which is less commonly associated with human-to-human transmission [13].

The incubation period of monkeypox is typically from 6 to 16 days (range: 5–21) [2,9]. The symptoms, which are usually self-limiting, are divided into an invasion (fever, lymphadenopathy, myalgia) and eruption phase. Monkeypox produces characteristic lesions that are typical of orthopoxviruses. Lymphadenopathy is a key feature that can help differentiate monkeypox from diseases with similar initial presentation (e.g. chickenpox, measles, smallpox) [2]. Skin eruption usually starts within 1 to 3 days of onset of fever and is usually localised to the face and extremities, with mucous membranes, genitalia, and palms/soles also affected. The rash develops sequentially from macules to papules to vesicles and finally to pustules which crust, dry and fall off. Lesion quantity ranges from a few to many thousands. These dry lesion crusts present a potential transmission risk. Case fatality rates range from 1% to 11% and are highest with the Central African clade and/or younger children [14].

There is no vaccine currently licensed for use specifically against monkeypox. Observational trials have shown that smallpox vaccines provide up to 85% protection against monkeypox [2]. More people will become susceptible to monkeypox as the proportion vaccinated against smallpox declines due to the removal of routine vaccination [15]. A third generation of smallpox (MVA-BN/Imvanex) vaccine has been approved for pre- and post-exposure prophylaxis for the prevention of smallpox and monkeypox and antiviral drugs are being developed [2].

The detection and control of this outbreak of imported monkeypox was aided by concurrent COVID-19 travel isolation requirements and the control measures in the emergency department, which limited the number of potential contacts. A coordinated multiagency response facilitated the prolonged self-isolation of the family in a safe and secure manner. Successful contact tracing of the 38 individuals by public health teams ensured any potential new cases would have been identified, in this instance there were no new cases outside the household. Hospital infection, prevention and control measures prevented onward transmission to healthcare workers.

## Conclusion

These and other recently imported cases reinforce the importance of infectious disease surveillance, including detailed travel and activity histories, which is critical for the implementation of an appropriate public health response. Incident response is aided by the rapid activation and expertise of the HCID network, with prompt sharing of information with the WHO in accordance with the IHR. COVID-19 control measures limited the number of potential contacts and secondary transmission occurred within the family only. The toddler is the first paediatric case outside Africa since the US outbreak in 2003.

## References

[1] Foster SO Brink EW Hutchins DL Pifer JM Lourie B Moser CR Human monkeypox. Bull World Health Organ. 1972;46(5):569-76. 4340216 PMC2480784

[2] World Health Organization (WHO). Monkeypox key facts. Geneva: WHO; 2019. Available from: https://www.who.int/news-room/fact-sheets/detail/monkeypox

[3] World Health Organization (WHO). Disease outbreak news: Monkeypox - the United States of America. Geneva: WHO; 2021. Available from: https://www.who.int/emergencies/disease-outbreak-news/item/monkeypox---the-united-states-of-america

[4] Department for Transport and Department of Health and Social Care. Guidance: Red, amber and green list rules for entering England. London: GOV.UK; 2021. Available from: https://www.gov.uk/guidance/red-amber-and-green-list-rules-for-entering-england#history

[5] Public Health England (PHE). COVID-19: guidance for maintaining services within health and care settings. Infection prevention and control recommendations version 1.2. London: PHE; 2021. Available from: https://www.gov.uk/government/publications/wuhan-novel-coronavirus-infection-prevention-and-control#history

[6] World Health Organization (WHO). International health regulations. Geneva: WHO; 2005. Available from: http://www.who.int/ihr/publications/9789241596664/en/

[7] Public Health England (PHE). Guidance monkeypox. The epidemiology, symptoms, diagnosis and management of monkeypox virus infections. London: PHE; 2018. Available from: https://www.gov.uk/guidance/monkeypox

[8] Nigeria Centre for Disease Control (NCDC). Monkeypox factsheet. Abuja: NCDC; 2019. Available from: https://ncdc.gov.ng/diseases/info/M

[9] Reynolds MG Yorita KL Kuehnert MJ Davidson WB Huhn GD Holman RC Clinical manifestations of human monkeypox influenced by route of infection. J Infect Dis. 2006;194(6):773-80. 10.1086/505880 16941343

[10] Nigeria Centre for Disease Control (NCDC). An update of monkeypox outbreak in Nigeria for week 21. Abuja: NCDC; 2021. Available from: https://ncdc.gov.ng/diseases/sitreps/?cat=8&name=An%20Update%20of%20Monkeypox%20Outbreak%20in%20Nigeria

[11] European Centre for Disease Prevention and Control (ECDC). Monekypox factsheet. Stockholm: ECDC; 2019. Available from: https://www.ecdc.europa.eu/en/all-topics-z/monkeypox/factsheet-health-professionals

[12] Petersen E Kantele A Koopmans M Asogun D Yinka-Ogunleye A Ihekweazu C Human monkeypox: epidemiologic and clinical characteristics, diagnosis, and prevention. Infect Dis Clin North Am. 2019;33(4):1027-43. 10.1016/j.idc.2019.03.001 30981594 PMC9533922

[13] Yinka-Ogunleye A Aruna O Ogoina D Aworabhi N Eteng W Badaru S Reemergence of human monkeypox in Nigeria, 2017. Emerg Infect Dis. 2018;24(6):1149-51. 10.3201/eid2406.180017 29619921 PMC6004876

[14] Beer EM Rao VB. A systematic review of the epidemiology of human monkeypox outbreaks and implications for outbreak strategy. PLoS Negl Trop Dis. 2019;13(10):e0007791. 10.1371/journal.pntd.0007791 31618206 PMC6816577

[15] Nguyen PY Ajisegiri WS Costantino V Chughtai AA MacIntyre CR. Reemergence of human monkeypox and declining population immunity in the context of urbanization, Nigeria, 2017-2020. Emerg Infect Dis. 2021;27(4):1007-14. 10.3201/eid2704.203569 33756100 PMC8007331

